# The Alcohol Mixed with Energy Drink Debate: Masking the Facts! A Commentary on “Mixing an Energy Drink with an Alcoholic Beverage Increases Motivation for More Alcohol in College Students” by Marczinski and Colleagues (in press)

**DOI:** 10.1111/acer.12004

**Published:** 2012-10-18

**Authors:** Joris C Verster, Chris Alford, Andrew Scholey

**Affiliations:** Division of Pharmacology, Utrecht Institute for Pharmaceutical Sciences, Utrecht UniversityUtrecht, The Netherlands; Psychology Department, Faculty of Health and Life Sciences, University of the West of EnglandBristol, United Kingdom; Centre for Human Psychopharmacology, Swinburne UniversityMelbourne, Victoria, Australia

To the Editor:

In their study “Mixing an Energy Drink with an Alcoholic Beverage Increases Motivation for More Alcohol in College Students,” Marczinski and colleagues (in press) conclude that “An energy drink may elicit increased alcohol priming. This study provides laboratory evidence that AmED beverages may lead to greater motivation to drink versus the same amount of alcohol consume alone.” However, on inspection of the study, we believe that this conclusion is premature and that issues with the design, analysis, and interpretation of the data may merit different conclusions.

The first, less serious issue relates to the nature of the vehicle, Squirt, which is described by the manufacturers as a caffeine-free citrus soda. If we assume that the participants had some knowledge of Red Bull, then the sensory characteristics of the vehicle and alcohol compared with energy drink and alcohol mixed with energy drink (AmED) may have produced different expectancies, potentially confounding the main outcomes of the study.

The second, more substantive issue relates to the analyses performed. The study utilized a between-subjects design and compared the effects of a placebo (Squirt lemon drink), alcohol, Red Bull energy drink, and AmED. The study includes several subjective measures but focuses on “desire to drink” which was assessed at baseline then at 10, 20, 40, 60, and 80-minutes postdrink. These scores were appropriately subjected to a 2 (Alcohol) × 2 (Energy Drink) × 2 (Gender) × 6 (Time) analysis of variance with time as a repeated measures factor. This revealed a significant interaction (*p* = 0.046). An appropriate analysis would then be to compare treatment groups at each time point which the authors then did using independent samples *t*-tests comparing alcohol and AmED groups. The authors state “No differences between alcohol and AmED for any of the time points were observed, although there was a nonsignificant trend for higher ratings for AmED versus alcohol alone at 40 minutes, *t*(38) = 1.47, *p* = 0.075.” The between-groups comparison, therefore, failed to reveal any significant effects.

In addition to the above, the authors compared, within each group, each time point with their respective baseline scores using paired samples *t*-tests. Unfortunately, this analysis tells us nothing about the differences between groups (Nieuwenhuis et al., 2011), for example, whether there are any differences between the alcohol and the AmED groups. However, they have used the results of the within-groups analysis to make inferences about differences between groups, even though they have shown these to be nonsignificant. This same analytical approach was used by some of these authors in an earlier study which then also drew erroneous conclusions (Marczinski et al., 2011). Additionally, such analyses can be greatly influenced by chance baseline differences in the same measure and by regression to the mean. Indeed, inspection of baseline “desire for alcohol” ratings (table 1) reveals large variations in mean baseline scores (energy drink 13.00, vehicle 7.80, alcohol 6.65, and AmED 4.65). This reflects sampling variability associated with the study and provides a range estimate (8 units) with which to compare posttreatment differences. From the graph (fig. 1), the maximum differences between alcohol and AmED appear to be of a similar magnitude supporting the lack of significant differences found.

These baseline scores directly predict the magnitude (and duration) of apparent change from baseline scores. Thus, using their questionable analyses, the authors report that consumption of Red Bull alone (with the highest baseline score) *reduced* “desire for alcohol” at 40 and 80 minutes. The alcohol drink increased “desire for alcohol” at 10 and 20 minutes, while the AmED with the lowest baseline score resulted in increased rating, relative to baseline at 10, 20, 40, and 80 minutes. It is notable that the consumption of vehicle alone, with the third lowest baseline score, resulted in significantly higher “desire for alcohol” restricted to 10 minutes postdrink—surely the most remarkable result of the study! Indeed, if these data are taken at face value then surely the fact that drinking citrus-flavored soda primes alcohol craving at all would be the cause of a major health concern. Of course a more realistic interpretation would compare scores between groups and conclude that there are no effects.

Despite the fact that there were no significant between-group differences for this measure, at several points in the discussion section the authors state “we observed that a priming dose of AmED increased these desire ratings for a longer time period compared with a priming dose of alcohol alone,” and “…, participants desired more alcohol following AmED compared with alcohol alone, although they liked and felt the 2 types of alcoholic beverages in a similar fashion.”

In the discussion section, Marczinski and colleagues (in press) characterize their results as “remarkable” because “Previous research suggests that this amount of caffeine alone would not reliably alter physiological or subjective state” (i.e., typically 46 mg caffeine). Indeed, it has been consistently shown that coadministering caffeine (<300 mg) generally does not significantly alter performance impairment caused by alcohol, nor do these levels of caffeine alter mood, or perception of intoxication (for a review, see Verster et al., 2012). Based on this empirical evidence, caffeine does not generally counteract the sedative effects of alcohol, and the nonsignificant results of the current study confirm previous findings. Nevertheless, Marczinski and colleagues (in press) interpret their data as suggesting that “…the energy drink mixer might increase the reinforcing aspect of alcohol.”

There is a current fashion to attribute “high risk” to mixing alcohol with energy drinks, in the face of data which does not support such a contention. In fact, findings from Marczinski and others consistently demonstrate that mixing alcohol with energy drink does not increase the desire to drink more (Marczinski et al., in press) and does not alter the perception of subjective intoxication (Alford et al., 2012; Attwood et al., 2012; Marczinski et al., 2011, in press).

Marczinski and colleagues (in press) continue the discussion by stating “Considering that drug wanting (i.e., incentive salience) produces addictive behavior (Robinson and Berridge, 1993), the results of our study might therefore provide an explanation for why consumers of AmEDs are more likely to become alcohol-dependent (Arria et al., 2011).” Again, this hypothesis is not supported by the data presented by Marczinski and colleagues (in press) in which drug “wanting” (as observed in craving) was not assessed. Instead, it was simply asked whether participants had a greater desire for alcohol after AmED compared with consuming alcohol alone. The results showed that this was not the case. Second, no significant differences were observed in “feeling” the beverage (whatever this means) and “liking” the beverage between AmED and alcohol only. Therefore, none of these subjective measures significantly distinguished between the alcohol and AmED treatments. The data presented by Marczinski and colleagues (in press) do not provide any evidence for the proposed relationship between AmED consumption and the risk of becoming alcohol-dependent (Arria et al., 2011), a relationship that has been disputed by several other researchers (Verster and Alford, 2011; Verster et al., 2012) and described as an “imaginary link” (Skeen and Glenn, 2011).

In conclusion, the data do not support the proposal that “The results of the current study suggest that increased translational research may better elucidate possible underlying mechanisms explaining why AmEDs may lead to increased drinking.” Marczinski and colleagues (in press) have confirmed that the desire to drink more was not significantly different after consuming AmED compared with consuming alcohol alone. Moreover, it was never assessed whether these subjects actually drink more after consuming AmED.

Finally, grant awarding agencies such as NIAAA should critically examine research proposals in this field to determine if the assumptions are based on scientifically verifiable facts. A succession of studies and surveys on AmED consumption has been shown to be of poor methodological quality which has resulted in a series of unjustified concerns and wrong conclusions (Verster et al., 2012). While there might be justifiable concerns, these need to be reinforced with high quality empirical data, and appropriate analyses. Presenting results without reference to the conventions of scientific reporting reduces the credibility of the alcohol research community as a whole.
